# Use of a social media network to reduce early neonatal mortality: a preliminary report from a quality improvement project in Yaoundé, Cameroon

**DOI:** 10.1186/s40748-017-0064-y

**Published:** 2017-11-13

**Authors:** Adidja Amani, Jobert Richie Nansseu, Evelyn M. Mah, Clemence Meguejio Vougmo, Seidou Moluh Moluh, Robinson Mbu

**Affiliations:** 1 Department of Family Health, Ministry of Public Health, Yaoundé, Cameroon; 2 Department for the Control of Disease, Epidemics and Pandemics, Ministry of Public Health, Yaoundé, Cameroon; 3 Department of Public Health, Faculty of Medicine and Biomedical Sciences of the University of Yaoundé I, PO Box 1364, Yaoundé, Cameroon; 4 Neonatology Unit, Yaoundé Gyneco-Obstetric and Pediatric Hospital, Yaoundé, Cameroon; 5 Department of Pediatrics and Specialties, Faculty of Medicine and Biomedical Sciences of the University of Yaoundé I, Yaoundé, Cameroon; 6 Neonatology Unit, Mother and Child Centre of the Chantal Biya Foundation, Yaoundé, Cameroon; 7 Department of Gynecology, Obstetrics and Specialties, Faculty of Medicine and Biomedical Sciences of the University of Yaoundé I, Yaoundé, Cameroon

**Keywords:** Perinatal network, Neonatal mortality, WhatsApp, Yaoundé, Cameroon

## Abstract

**Background:**

Perinatal networks have yielded substantial contribution in decreasing the neonatal mortality rate. We present here the process of implementation of a perinatal network in Yaoundé (Cameroon) based on the WhatsApp messenger application as well as some preliminary results and achievements.

**Methods:**

In December 2016, the Yaoundé Perinatal Network was launched, regrouping a multidisciplinary team of health professionals dealing with perinatal care in Yaoundé, Cameroon. The network takes advantage of WhatsApp facilities and is coordinated by 5 administrators. One of their main duties is to have a twice-daily updated status of the available equipment (incubators, oxygen and phototherapy) and bed capacities across the Yaoundé pediatric units. Once a request is sent through the network, other members react, either by giving advice or by telling where the desired equipment or expertise is available at that moment. Then, the baby is immediately prepared for transfer, occurring once the receiving pediatric unit has attested that it is already prepared to receive the new patient.

**Results:**

From December 18, 2016 to July 31, 2017, 139 members representing all the principal maternities and tertiary pediatric units in Yaoundé were already included in the network. The network permitted instant sharing of knowledge and information between its members for an optimal delivery of care. Two hundred and seventeen neonates were transferred using the network; the median time of response after a request had been sent was 19.5 min and the delay in transferring a neonate averaged 70 min.

**Conclusion:**

Taking account of the preliminary promising notes, there is hope that the Yaoundé Perinatal Network will help to reduce neonatal mortality in our context. Lessons learned from its implementation will serve to replicate this innovative health action in other towns of the country. Moreover, this experience could be a source of inspiration for other countries facing similar challenges.

## Background

Maternal and child mortalities remain very preoccupying worldwide, especially in economically-deprived areas. Indeed, according to World Health Organization estimates for 2015, 303,000 women died due to pregnancy or childbirth-related complications, while 5.6 million of children aged below 5 years old died in 2016, 45% of these deaths occurring in the first 28 days of life [1, 2]. Almost 80% of deaths of children aged under 5 years occur in sub-Saharan Africa or Southern Asia [3]. In Cameroon specifically, 22,542 neonatal deaths occurred in 2013, with a neonatal mortality rate per 1000 live births of 28.6 [4]. The reduction in neonatal mortality has been set among the priorities for each and every country to at least as low as 12/1000 live births [3].

Seventy-five percent of neonatal deaths occur during the first week of life, of which 25–45% arise within the first 24 h [5]. The main causes of these deaths include prematurity and low-birth-weight, infections, asphyxia and birth trauma; they are responsible for almost 80% of deaths in this age group [5]. Consequently, up to two-thirds of neonatal deaths could be prevented if effective health measures were provided at birth and during the first week of life. In developing countries where the large majority of neonatal deaths still occur, access to health care remains very low, with nearly half of all mothers and babies who do not receive adequate care during and immediately after birth [5].

In Cameroon, a sub-Saharan African and lower medium class country, the newborn referral system from one health facility to another one having sufficient and/or adequate equipments (oxygen, incubators, and/or phototherapy for example) is practically inexistent. Consequently, parents are those who transport their newborns from one hospital to another, obviously in bad conditions and sometimes without eventually finding where the newborn will be admitted, especially when he/she is a premature baby. The newborn will die in parents’ hands, or reach the hospital in a really bad health condition, which will considerably hamper his/her chances of survival. Indeed, it has been shown that the mortality rate among babies transferred is higher compared to those born in the health facility, this being truer for premature neonates [6]. It is therefore high time urgent, realistic, cheap and meaningful strategies be put in place to create a perinatal network which will boost our newborn referral system, with the ultimate goal of improving early neonatal survival rates in Cameroon. In this line, the global use of mobile devices with their connectivity capacity, and integrated with the social media networks, provides a resource-rich platform for innovative health actions. We present here the process of implementation of a perinatal network in Yaoundé (Cameroon) based on the WhatsApp messenger application with some preliminary results and achievements.

## Methods

### Setting up the network

A meeting was organized in Yaoundé, capital city of Cameroon, as part of activities marking the celebration of the Maternal and Neonatal Health week held in June 2016. This meeting was placed under the leadership of the Ministry of Public Health (MoH), and convened maternal and newborn health professionals from public, private and faith-based health facilities located in Douala, Nkongsamba and Yaoundé, Cameroon. It was agreed during this session that our newborn referral system is very embryonic and ineffective, leading to many early neonatal deaths especially among premature babies [6]. One of the foremost resolutions was the establishment of a perinatal network which could be of substantial help in ameliorating this referral system. The mandate of this network would be to improve the quality care through a perinatal referral system of neonates towards health facilities with adequate and functional equipments (such as incubators, oxygen and phototherapy) as well as experienced and specialized workforce. As a consequence, this network would contribute significantly in reducing early neonatal mortality rates in our context as shown elsewhere [7].

On the 17th December 2016, a perinatal transfer platform was created for the city of Yaoundé called the “Yaoundé Perinatal Network”. Considering that nowadays each and every person has a smartphone and can easily get data bundles for a non-stop reliable internet connection, this perinatal network took advantage of a widespread used social media networking: the WhatsApp messenger application.

### Requirements for the network

The network is based on the WhatsApp application which is an instant messaging service. It requires for each potential member of the network to have a smartphone in which the WhatsApp application has been downloaded and installed. The other requirement is to have a reliable internet connection available 24 h/24. Currently in Cameroon, 5 mobile phone companies do offer fairly reliable internet connections, with data bundles for WhatsApp that can be accessed at cheaper prices. WhatsApp was chosen as the network pedestal taking into account its advantages (convenience, user-friendliness, simplicity and high connectivity) which will facilitate instant communication and interactions within the network. Additionally, use of this social media networking is widespread across the country and it has gained much popularity in comparison to other applications which are less used. The choice of WhatsApp was not based on any relation, commercially or not, to the WhatsApp application holders.

### Elements of the network

A team made up of 5 administrators coordinates the network, including 2 pediatricians, 1 obstetrician and two officials of the Department of Family Health at the MoH, Cameroon. They are responsible for seeking and adding new members to the network. In addition, these administrators have other fundamental roles, comprising: (i) emphasis on the clarity, preciseness and conciseness of messages to be shared; (ii) reminders on the importance of sending messages only related to newborns such as availability of incubators, phototherapy, oxygen and/or bed space for neonates, conditions for safe transfer of the newborn(s), advice for urgent management while waiting for the transfer; (iii) briefing of the new comers on the objectives of the network and types of messages to be shared in order to avoid any deviation. The lead administrator of the network, who is one of the 2 officials at the MoH, has the responsibility of requesting from members working in reference pediatric units to make an update on the current status of functional equipments and hospital capacities in their units; this is done twice-daily.

To be a member of the network, the person must be a health professional working in a health facility located in Yaoundé, and must be dealing with perinatal care. In this regard, the network members are from a multidisciplinary background including specialized obstetricians and pediatricians, residents in obstetrics, residents in pediatrics, midwives, pediatric surgeons, pediatric cardiologists, neurosurgeons, and heads of maternity and pediatric units. New members are identified on a regular basis by administrators of the network using a snowball technique; they are helped by other members who can suggest to include their colleagues in the network. Members are subjected to exclusion from the network if their postings recurrently tend to deviate from the network’s objectives.

### Coverage of the network

The network is expected to cover all the maternities and pediatric units of Yaoundé by including health professionals working in these units.

### Functionality of the network

Upon need, a member of the network immediately sends a message, briefly presenting his/her patient and expressing the urgent need for the newborn. The application enables each member of the network to receive an alert each and every time a message is sent through the network. The other members react, either by giving advice or by telling where the desired equipment or expertise is available. Once the information is available, the baby is transferred, and the receiving hospital already knows that a baby is arriving and starts preparing accordingly. The member(s) of the receiving health facility is (are) required to give a feedback on the health status and outcome of babies transferred through the network.

Importantly, members of the network are required to send details of the current neonatal hospital capacity and available functional equipment in their health facility (incubators, oxygen, and phototherapy). Twice-daily, the lead administrator of the network is in charge of remembering the members in this respect or requesting this information from members.

### Referral of newborns

Women with complicated pregnancies are referred before delivery, or babies at birth. The receiving health facility is alerted of a new arrival, and gives its “Okay” before the baby is transported from the transferring health facility to the receiving one. Stable newborns are transported using the Kangaroo method.

### Data extraction and analysis

In order to present our preliminary achievements, data were pulled out from all messages sent through the network between December 18, 2016 and July 31, 2017. The number, types and purposes of messages were reported. Additionally, we retrieved information on the time elapsed between a request and a response, reasons for transfer, and the turnaround time for transfer. On the other hand, fluidity of communication between members of the network was evaluated. Two tools were used for computation and online visualization of WhatsApp messages (http://chatvisualizer.com and © WhatsAnalyzer – Julius-Maximilians-Universität Würzburg whatsanalyzer.informatik.uni-wuerzburg.de). Subsequently, data were coded, entered and analyzed using Microsoft Excel v. 2013 for Windows. Descriptive statistics were used to summarize our preliminary results.

### Preliminary results

#### Members of the network

On July 31st 2017, 139 health professionals were members of the Yaoundé Perinatal Network. All these members were working in categories 1 (General Hospitals), 2 (Central Hospitals), and 4 (District Hospitals) health facilities located in Yaoundé, Cameroon.

### Activity within the network

**Fig. 1:**
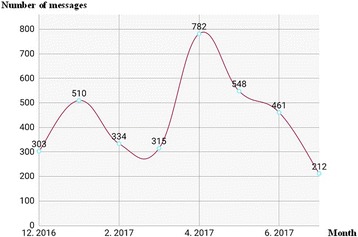
Number of messages per month, from December 2016 to July 2017

**Fig. 2:**
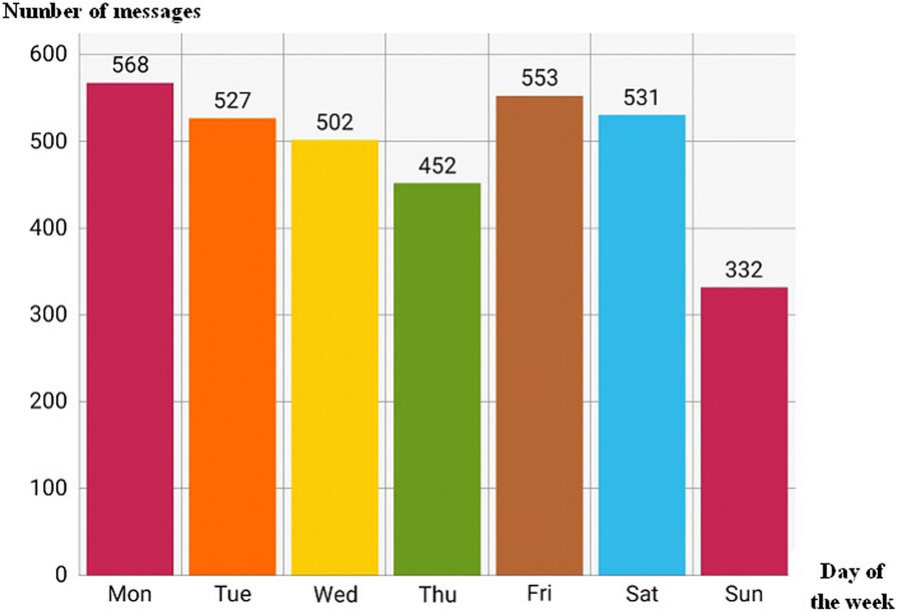
Total number of messages shared within the network, per each day of the week

**Fig. 3:**
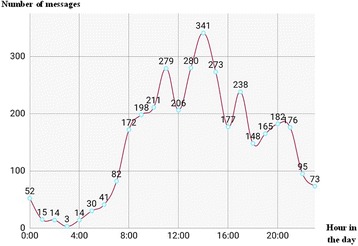
Number of messages shared in the network presented according to different hours in a day

A total of 3453 messages were shared within the network from December 2016 to July 2017, with an average of 15 messages per day. A maximal number of 782 messages were shared during the month of April 2017 (Fig. 1). Activity of the network was maximal on Fridays and Mondays (Fig. 2); the most reported period of chatting was between 11 am and 3 pm, with a pic at 2 pm (Fig. 3). By contrast, the least period of texting was between 1 and 4 am (Fig. 3). The types of postings were mostly messages; 62 pictures were shared, and audio- and video-postings were not used at all. About 92.7% of messages were directly related to neonatal patients’ care. The network enabled each of its members to have rapid and timely access to a load of information for a better management of newborn patients and their mothers.

### Reactivity and efficacy of the network

During the same period, 217 patients were transferred from one health facility to another, all of which were included in the network. The majority of patients were premature babies (109/217; 50.2%); 105 babies (48.4%) were born at term, 2 babies (0.9%) were born after term and one baby (0.5%) was small for gestational age. All transfers occurred from a maternity unit to a reference pediatric unit having the required need or expertise (equipment and/or specialized personnel). The main reasons for transfer included the need for an incubator because of prematurity (68/217; 31.3%), the need for oxygen because of neonatal asphyxia (46/217; 21.2%), infection (30/217; 13.8%), the need for phototherapy (13/217; 6.0%), and congenital malformations (3/217; 1.4%). Two deaths were recorded: one case of neonatal meningitis and one very preterm baby.

Instant sharing of adequate and updated information enabled all members of the network to be efficient towards the management of their patients. The median time for response following a request was 19.5 min. The average transfer time was 70 min; 60% of newborns were transferred within the first 6 h of life and 22% within 2 h. About 105 transfers (48.4%) were performed in the afternoon, especially between 1 pm and 3 pm.

## Discussion

The current context is marked by a crucial shortage of up-to-date pediatric units, adequate equipment and specialized workforce on one hand [5, 8], and a high neonatal mortality rate especially among premature babies and those transferred on the other hand [6]. Therefore, implementation of a perinatal network could be of substantial contribution in improving our newborn referral system with a consequential reduction in early neonatal mortality as it was observed elsewhere [7]. This was the rationale for creating a perinatal network in Yaoundé, Cameroon. This paper purposed to share our experience of using the WhatsApp application as the platform for this network, and present some preliminary achievements in less than 8 months.

The network is in gradual expansion. In not more than 8 months, 139 members have already been enrolled. The network has permitted to connect all the principal maternity units to all the tertiary care pediatric units located in Yaoundé. However, only 217 babies were transferred through the network, suggesting that we should communicate more on this new platform. Notwithstanding, we noticed a great improvement over time. In fact, until April 2017, only 61 babies have been transferred through the network. Health professionals dealing with pediatric care in Yaoundé should be continuously sensitized and briefed on the public health benefits of participating in such a network; new techniques need to be found in order to include new members and motivate all adherents for better implication and participation.

Our objective is that each and every health facility in Yaoundé be represented in this perinatal network. Moreover, lessons learned from this pioneer experience will serve to establish similar networks in other towns of the country and connect them to each other. Further, considering that our global referral system is very weak, this kind of networks could benefit other fields of great public health impact such as maternal health and blood transfusion. Current evidence shows indeed that the problem of blood transfusion remains a big challenge in sub-Saharan African countries [9]. This experience could also be replicated in other countries facing similar challenges.

Even though we do not have baseline data for comparisons, our preliminary results are somehow indicative of promising changes towards early neonatal care. The network permitted to deliver adequate care as soon as possible, minimizing therefore inconveniences from patients and their families. It is true however that the time elapsed between the moment a request was sent and the moment a response was given has to be shortened, as well as the transfer time. For the former concern, we need to think about how a number of designated members could seek the right information and react to a request as soon as it has been sent. Concerning the latter issue, we believe that a system for patient transportation needs to be developed if we want to shorten the delay in transferring our patients from one health facility to another. From another perspective, we should seek appropriate measures to address the low activity within the network at night.

The Yaoundé perinatal network takes advantage of the friendliness and high connectivity of the WhatsApp application which is freely accessible, cheap and very easy to use. As a consequence, there is instant exchange of capital information within the network, enabling its members to deliver adequate care towards their patients. However, there are some drawbacks which can dreadfully hamper the network’s functioning. First, it is required to have a smartphone, meaning that if a person does not have a smartphone, he/she cannot enter the network. Another difficulty with smartphones is their battery: the phone’s energy does not last for long, especially when the phone is connected to the internet; as a consequence, the phone needs to be charged regularly. This means that a reliable source of electricity should be always available, in a context of recurrent electricity shedding. Second, running the WhatsApp application supposes a reliable and stable internet connection, which is not always the case in every corner of our towns despite presence of five mobile phone companies in Cameroon. Furthermore, even though data bundles for WhatsApp are cheap, it is the responsibility of every member of the network to buy data bundles for his/her phone, something capable of destabilizing the network or impeding its performances. The MoH alongside its partners could think of supporting this initiative, by sending data bundles for WhatsApp in each member’s phone on a regular basis. Moreover, a qualitative analysis of how health professionals and even the general public perceive the potential advantages and limitations of the network along with their suggestions is warranted to come-up with a stronger perinatal network which will surely result in bending the neonatal mortality rate.

## Conclusion

Taking into account these preliminary promising notes, there is hope that the Yaoundé Perinatal Network will help to reduce neonatal mortality in our context. Lessons learned from its implementation will serve to scale up this innovative health action in other towns of the country. Other fields of intervention could also benefit from this initiative which could be replicated in other countries facing similar challenges. Our Governments should take advantage of this innovative approach, and offer its support to the system for long lasting outcomes. Moreover, the network weaknesses should be rapidly identified and addressed accordingly.

## Abbreviation

MoH: Ministry of Public Health (Cameroon)

## References

[1] World Health Organization: Media Centre - Maternal mortality - Fact sheet. 2016. http://www.who.int/mediacentre/factsheets/fs348/en/. Accessed 25 Oct 2017.

[2] World Health Organization: Media Centre - Children: reducing mortality - Fact sheet. 2017. http://www.who.int/mediacentre/factsheets/fs178/en/. Accessed 25 Oct 2017.

[3] United Nations: Sustainable Development Goals, 17 goals to transform our world - Goal 3: Ensure healthy lives and promote well-being for all at all ages. 2017. http://www.un.org/sustainabledevelopment/health/. Accessed 25 Oct 2017.

[4] WHO | Country profiles on neonatal and child health [Internet]. 2017 [cited 2017 Feb 5]. Available from: http://www.who.int/maternal_child_adolescent/epidemiology/profiles/neonatal_child/en/#C.

[5] World Health Organization: Media Centre - Newborns: reducing mortality - Fact sheet. 2017. http://www.who.int/mediacentre/factsheets/fs333/en/. Accessed 25 Oct 2017.

[6] Njom Nlend AE, Zeudja C, Ndiang S, Nga Motaze A, Ngassam Laurence L, Nsoa L (2015). Mortality in a neonatology unit in 2013 in Yaounde (Cameroon): rationale for a perinatal network. Arch Pediatr.

[7] Burguet A, Di Maio M, Besnier-Di Maio S, Kayemba-Kay’s S, Nassimi A, Bouthet M-F (2007). Very preterm birth less than 33 weeks’ gestation: how setting-up of a perinatal network does influence the activity of the neonatal tertiary care unit? The experience of the Poitou-Charentes region, France. J Gynecol Obstet Biol Reprod (Paris).

[8] Nansseu JR, Bigna JJ (2017). Antiretroviral therapy related adverse effects: can sub-Saharan Africa cope with the new “test and treat” policy of the World Health Organization?. Infect Dis Poverty.

[9] Nansseu JR, Noubiap JJ, Ndoula ST, Zeh AF, Monamele CG (2013). What is the best strategy for the prevention of transfusion-transmitted malaria in sub-Saharan African countries where malaria is endemic?. Malar J.

